# Tumor volume is an independent predictor of survival in patients with malignant pleural mesothelioma

**DOI:** 10.1186/1749-8090-10-S1-A48

**Published:** 2015-12-16

**Authors:** Diana Y Kircheva, Aliya Husain, Sydeaka Watso, Samuel Armato, Hedy Kindler, Wickii T Vigneswaran

**Affiliations:** 1 Department of Surgery, University of Chicago Medicine, Chicago, IL 60637, USA; 2 Department of Pathology, University of Chicago Medicine, Chicago, IL 60637, USA; 3 Department of Biostatistics, University of Chicago Medicine, Chicago, IL 60637, USA; 4 Department of Radiology, University of Chicago Medicine, Chicago, IL 60637, USA; 5 Department of Medicine, University of Chicago Medicine, Chicago, IL 60637, USA

## Background/Introduction

Tumor histology and stage are predictors of survival in patients with malignant pleural mesothelioma (MPM). The TNM classification that is widely accepted for MPM has its limitations. Significance of tumor volume, a better representation of tumor burden is not routinely determined. We hypothesized tumor volume is a better predictor of survival and complimentary to T and N stage. Extended pleurectomy and decortication (EPD), a lung sparing procedure, provides an opportunity to measure the tumor volume and pathological stage.

## Aims/Objectives

Evaluate the significance of tumor volume on overall survival in patients undergoing EPD for MPM.

## Method

111 patients who underwent EPD for MPM formed the basis of this report. The following variables were assessed: age, gender, histology including percent epithelioid histology and pathological T and N stage. Tumor volume of resected specimens was measured using a water displacement method. A Cox regression model was used to identify significant predictors of survival. Kaplan-Meier was used to summarize overall and subgroup survival.

## Results

There were 91 males and 20 females with a median age of 68 years (range 43-88 years). Median tumor volume was 560 ml (range 100-2200 ml). Tumor volume was less than 300 ml in 18 patients, between 301-600cc in 37 patients, 601-900 in 29 patients and >900cc in 25 patients. Five patients (4.5%) died within 30 days of surgery. Overall two year survival from diagnosis was 48.3% and from EPD was 31.5%. Tumor volume was a significant predictors of survival (p = 0.001, Table 1) and T stage (p = 0.05). No relationship between N stage and either tumor volume or histology was observed.

**Table 1:** 

Volume	Median survival
<300 ml	2.25 years
301-600 ml	1.62 years
601-900 ml	1.06 years
> 900 ml	0.9 years

## Discussion/Conclusion

Tumor volume is an independent predictor of survival in patients with MPM undergoing EPD.

Tumor volume is an important measure and is complimentary to TNM staging

